# Mercury Chalcogenide Colloidal Quantum Dots for Infrared Photodetectors

**DOI:** 10.3390/ma16237321

**Published:** 2023-11-24

**Authors:** Qun Hao, Haifei Ma, Xida Xing, Xin Tang, Zhipeng Wei, Xue Zhao, Menglu Chen

**Affiliations:** 1 Physics Department, Changchun University of Science and Technology, Changchun 130022, China; qhao@bit.edu.cn (Q.H.); xingxida@163.com (X.X.); xintang@bit.edu.cn (X.T.); weizp@cust.edu.cn (Z.W.); 2 School of Optics and Photonics, Beijing Institute of Technology, Beijing 100081, China; 3220220493@bit.edu.cn

**Keywords:** colloidal quantum dots, infrared photodetector, mercury chalcogenide

## Abstract

In recent years, mercury chalcogenide colloidal quantum dots (CQDs) have attracted widespread research interest due to their unique electronic structure and optical properties. Mercury chalcogenide CQDs demonstrate an exceptionally broad spectrum and tunable light response across the short-wave to long-wave infrared spectrum. Photodetectors based on mercury chalcogenide CQDs have attracted considerable attention due to their advantages, including solution processability, low manufacturing costs, and excellent compatibility with silicon substrates, which offers significant potential for applications in infrared detection and imaging. However, practical applications of mercury-chalcogenide-CQD-based photodetectors encounter several challenges, including material stability, morphology control, surface modification, and passivation issues. These challenges act as bottlenecks in further advancing the technology. This review article delves into three types of materials, providing detailed insights into the synthesis methods, control of physical properties, and device engineering aspects of mercury-chalcogenide-CQD-based infrared photodetectors. This systematic review aids researchers in gaining a better understanding of the current state of research and provides clear directions for future investigations.

## 1. Introduction

Extensive applications abound for infrared (IR) detection technology, spanning telecommunications, chemical spectroscopy, gas sensing, biomedical applications, military night vision, and autonomous driving [1,2,3,4,5,6,7,8,9,10,11]. However, the current commercial IR photodetectors are primarily based on epitaxially grown semiconductors such as InGaAs and HgCdTe [12,13,14,15,16,17,18,19,20]. Although these detectors offer advantages such as high sensitivity and good stability, they suffer from drawbacks including high manufacturing costs and incompatibility with silicon-based readout integrated circuits and are hindered by intricate epitaxial growth processes, which hinder further advancements in IR detection technology [21,22,23,24]. Building upon this, novel infrared detectors such as the quantum well IR photodetector [25,26,27,28,29,30], type-II superlattice [31,32,33,34,35], infrared detectors based on two-dimensional materials [36,37,38,39,40], and quantum dot IR photodetector [41,42,43,44,45,46,47,48] have begun to be extensively investigated.

Colloidal quantum dots (CQDs), as alternatives to traditional epitaxial-grown semiconductors, have emerged with unique advantages such as the ease of processing solutions, cost-effectiveness, scalability, and the ability to adjust size [49,50,51,52,53]. These advantages present a promising outlook for low-cost, large-scale, high-resolution, and small-pixel IR detectors. Over the past decade, CQDs have found widespread applications in various fields, including solar cells [54,55,56], spectroscopy [57], phototransistors [58,59,60], focal plane array (FPA) imagers [61], lasers [62,63,64], and light-emitting diodes [65,66]. Among them, mercury chalcogenide CQDs, due to their large Bohr radius and wide tunable size range, theoretically cover the critical IR spectral bands, positioning them as ideal materials for IR detectors that have the potential for superior detection performance [67,68]. Mercury chalcogenide CQDs encompass materials including HgTe, HgSe, and HgS CQDs. However, quantum dot photodetectors still face challenges, including material stability, surface modification, and difficulties in integrating with FPA [69].

This paper provides an overview of the recent progress on mercury chalcogenide CQD IR detectors and discusses the future directions for mercury chalcogenide CQDs in IR detection technology. Compared with other review articles [70,71], our discussion takes a detailed approach to three different materials and covers aspects such as material synthesis, optimization, and device fabrication. Additionally, we provide an in-depth analysis of the infrared detector structure based on HgTe CQDs, considering material synthesis, optimization, and device preparation. This comprehensive research approach makes an important contribution to the understanding of these materials in the context of current infrared detection technologies.

## 2. Research on HgTe CQDs

### 2.1. Synthesis of HgTe CQDs

Zero-gap HgTe quantum dots exhibit extensive potential applications owing to their elevated absorption coefficients, adjustable bandgaps spanning the entire infrared range, and distinctive optoelectronic characteristics [72,73,74].

In the late 1990s, Rogach et al. drew inspiration from their previous research on CdTe nanoparticles to propose an aqueous colloidal growth technique to synthesize HgTe CQDs [75,76,77]. They utilized 1-thioglycerol (1-mercaptopropane-2,3-diol) as the size-regulating capping agent. In the presence of thioglycerol as a ligand, mercury perchlorate was dissolved in water and, simultaneously, H_2_Te was bubbled into this solution as a source of Te^2−^. The inclusion of H_2_Te increased the complexity of the synthetic setup for aqueous synthesis. As a result, alternative methods for growth in organic media were subsequently developed.

**Figure 1:**
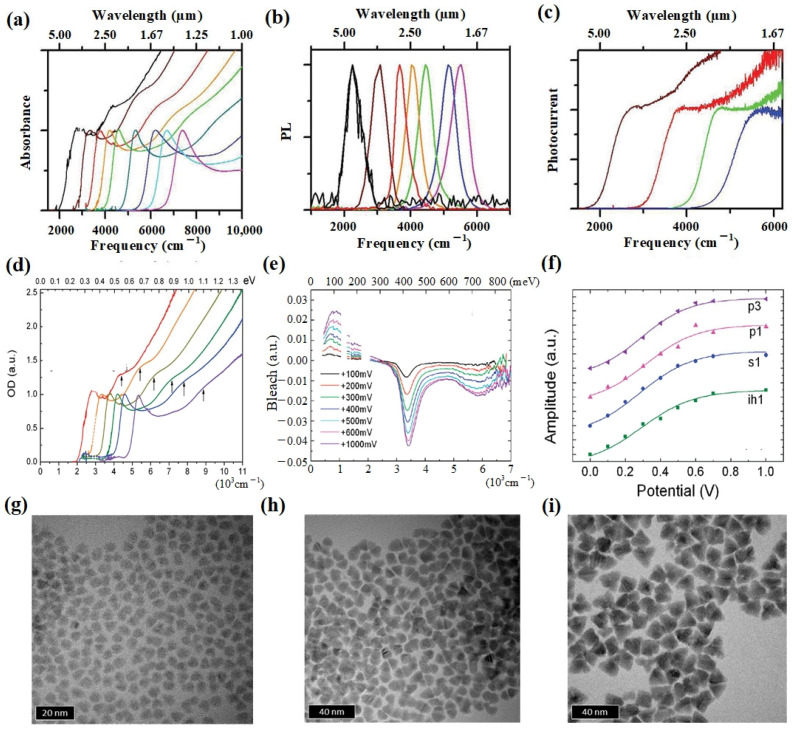
HgTe CQD Characterization. (**a**) Absorption spectra of HgTe CQDs of different sizes in C_2_Cl_4_ are presented, with the C-H absorbance from the ligands subtracted for clarity. (**b**) Photoluminescence (PL) spectra of HgTe CQDs of different sizes in C_2_Cl_4_. (**c**) Photoconduction spectra are presented for films composed of colloidal HgTe nanoparticles with varying sizes. The films exhibit typical optical densities of approximately 0.1 at the exciton and have an approximate thickness of 100 nm [78]. Copyright 2011, *J Am Chem Soc*. (**d**) The absorption spectra of HgTe CQDs of different sizes in tetrachloroethylene (TCE) solution at room temperature are depicted. A second feature is emphasized with vertical arrows. The region around the C-H stretch (2900 cm^−1^) has been excluded and substituted with dashed lines. (**e**) Differential spectra of a HgTe film recorded under positive potentials at 210 K is presented. Spectral regions where the electrolyte absorbs more than 70% have been excluded from the data. (**f**) The amplitude of the Gaussians corresponding to each transition as a function of the applied potential [84]. Copyright 2011, *The Journal of Physical Chemistry C*. (**g**–**i**) Transmission electron microscopy (TEM) images of particles with sizes of (**g**) 5.5, (**h**) 9.1, and (**i**) 15.5 nm [87]. Copyright 2014, *ACS Nano*.

In 2011, Keuleyan et al. successfully extended the absorption edge of HgTe CQDs to over 5 μm by improving the synthesis technology. They conducted a comprehensive discussion on the synthesis process of HgTe CQDs [78] (Figure 1a–c) and studied the optical properties [79], photoluminescence properties [80], and transport properties [81,82] of HgTe CQD films. These detailed studies provided a solid theoretical foundation for the design of HgTe CQD infrared detectors. They also first reported HgTe CQD photodetectors with a room-temperature photoresponse beyond 5 μm [83]. In the same year, Liu et al. discussed the electronic structure of HgTe CQDs [84] (Figure 1d–f). Employing liquid gating, they characterized the electrochemical response of HgTe CQD films, investigating the feasibility of achieving n-type and p-type charging and exploring the distinctions in electron and hole transport properties. They demonstrated electrochemical modulation of n-type and p-type carriers in HgTe CQD films. The results showed that although the carrier mobility of both n-type and p-type HgTe CQD films could reach 0.1 cm^2^/Vs, the p-type films demonstrated an increased photocurrent, whereas the n-type films displayed a more pronounced magnetoresistance effect.

In order to ensure the stable growth and good colloidal dispersion of CQDs, long-chain organic molecules are used as ligands during the synthesis stage of CQDs [85]. However, due to the influence of their own volume, substantial steric hindrance arises from the presence of elongated organic molecules with extensive chain lengths. Therefore, ligand exchange is often required during the preparation of CQDs to increase the coupling between CQDs via exchanging long-chain ligands for short-chain ligands in order to improve the carrier mobility of CQDs. Amine ligands exhibit low stability, making them prone to detachment during the centrifugal purification stage and thereby causing the aggregation of CQDs. On the other hand, thiol ligands form robust bonds with the Hg sites on the surface of HgTe CQDs, posing challenges for subsequent surface modifications [71]. Therefore, the selection of ligands and the improvement of the ligand-exchange technique are of paramount importance.

**Figure 2:**
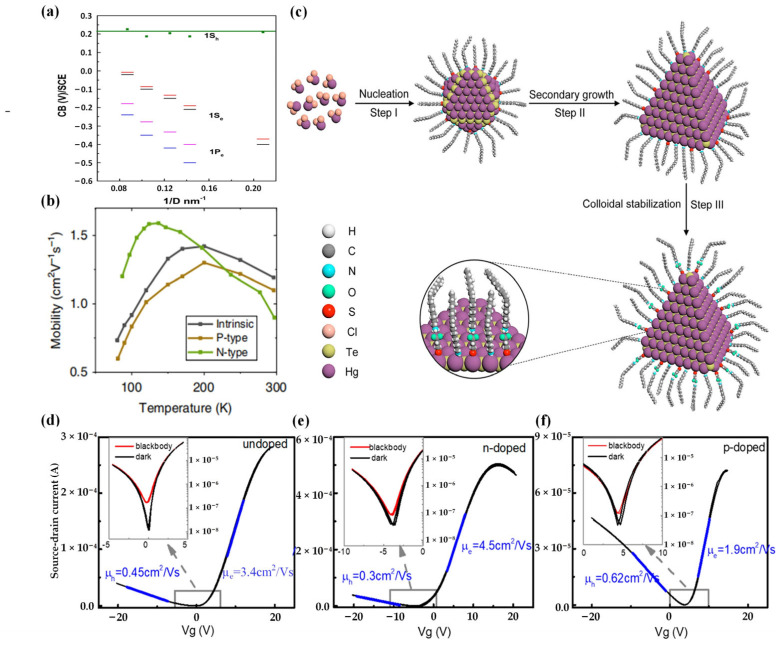
Ligand exchange on HgTe CQDs. (**a**) Energies of the states in HgTe synthesized using (trimethylsilyl)telluride (TMSTe) are plotted against size. The black and blue dashed lines represent the 1S_e_ and 1P_e_ states derived from electrochemistry, respectively. The red and magenta dashed lines depict the 1S_e_ and 1P_e_ states corrected by the charging energy. The green dots indicate the estimated conduction band minimum obtained from optical interband absorption [88]. Copyright 2017, *J Phys Chem Lett*. (**b**) Temperature-dependent mobility [91]. Copyright 2023, *Light Sci Appl*. (**c**) Visual representation of the controlled growth of HgTe CQDs through ligand engineering [93]. Copyright 2022, *Nano Lett*. (**d**–**f**) FET transfer characteristics at 80 K for near-intrinsic, n-doped, and p-doped HgTe/hybrid ligand films, respectively. Insets display enlarged regions near the conductance minima highlighting the photocurrent (red) and dark current (black). The blue lines represent the slopes utilized for calculating hole and electron mobility [89]. Copyright 2019, *ACS Photonics*.

In order to find suitable ligands, Lhuillier et al. explored the inorganic ligand exchange route in 2013, where they used arsenic sulfide (As_2_S_3_) to perform ligand exchange with HgTe CQD films [86]. The mobility of the As_2_S_3_-treated film was approximately 10^−2^ cm^2^/Vs, which was 100 times higher than that of the EDT-treated film. This study showed that the exchange and encapsulation of inorganic ligands could improve the electrical and optical properties of CQD films. In addition, using As_2_S_3_ solution to encapsulate CQDs could slow down the oxidation rate of CQD films in air. In order to enhance the stability of CQDs, Keuleyan et al. synthesized HgTe CQDs with high monodispersity by diluting the TOP (Te precursor with oleylamine), with a size up to 15 nm, corresponding to the room-temperature absorption edge at 5 μm [87] (Figure 1g–i). Diluted Te precursors could be fully mixed with HgCl_2_ solution to ensure a more uniform mixture before significant growth of CQDs occurred while maintaining a low enough temperature to sufficiently prevent additional nucleation. This made the size distribution of HgTe CQDs much improved. In 2017, Shen et al. mitigated the tendency of HgTe CQD aggregation by employing a more reactive tellurium source and an excess of mercury precursors. In addition, the size of the CQDs was adjustable from 4.8 to 11.5 nm [88] (Figure 2a). At the same time, the CQDs could be stably dispersed without mercaptan, which was conducive to further ligand exchange and the nuclear/shell structure growth of CQDs to improve the device performance.

For the improvement of ligand exchange technique, Chen et al. proposed a novel manufacturing process for HgTe CQD films in 2019 [89] (Figure 2d–f). They replaced the bulky oleylamine ligands with a combination of HgCl_2_, 2-mercaptoethanol, n-butylamine, and n-butylammonium chloride. This substitution resulted in the formation of stable nanoinks in N, N-dimethylformamide (DMF) and achieved high electron mobility in HgTe CQD films without compromising the discrete nature of the electronic states. During the ligand exchange process, the doping state of HgTe CQDs was controlled by adjusting the concentration of HgCl_2_. The optimized HgTe CQD films exhibited a high mobility up to 1 cm^2^/Vs. To further improve the mobility, in 2020 Lan et al. demonstrated that CQD solids can achieve high electron mobility without compromising the discrete nature of the electronic states [90]. They increased the mobility up to 8 cm^2^/Vs. Subsequently, Xue et al. further optimized the ligand exchange technique by introducing a mixed-phase ligand exchange method. This approach segregates mobility enhancement, doping control, and Fermi level adjustment into separate steps, providing precise control over the transport properties of CQDs [91] (Figure 2b).

Researchers are constantly exploring methods to ensure the stable growth and good colloidal dispersion of HgTe CQDs. In 2022, Liu et al. introduced an innovative method for synthesizing uniform mid-infrared HgTe CQDs and optimized the synthetic process of the mercury precursor solution [92]. They used HgI_2_ as the mercury source and broke the strong Hg-I bond by changing the reaction temperature. Finally, they achieved the successful in situ passivation of HgTe CQDs with I^−^, enabling tunable sizes ranging from 8 to 15 nm.

In addition, Yang et al. studied a ligand engineering method that could produce well-separated HgTe CQDs [93] (Figure 2c). Their strategy initially involved using strongly bound alkylthiol ligands to synthesize well-dispersed HgTe cores. This was succeeded by a secondary growth process and a final step of ligand modification to augment the colloidal stability. Using this method, the HgTe CQDs could be highly monodisperse in a large size range of 4.2~15.0 nm. The edge absorption was tunable in the wide infrared region of 1.7~6.3 μm, completely covering short-wave and mid-wave infrared regions. Moreover, electron mobility reached a record high of 18.4 cm^2^/Vs.

### 2.2. HgTe CQD Photodetectors

HgTe CQDs have received considerable attention in optoelectronic devices such as photoconductive devices, phototransistors, photovoltaic devices, and infrared focal plane array detectors due to their excellent electronic and optical properties, as well as controllable synthesis.

**Figure 3:**
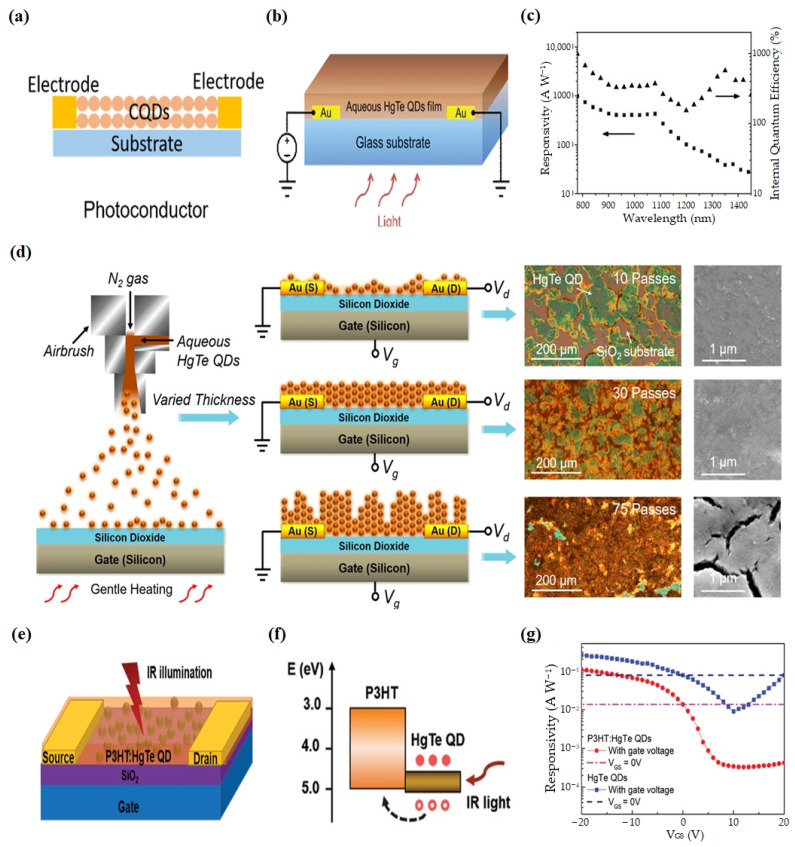
HgTe CQD photoconductive devices and phototransistors. (**a**) The typical device structure of photoconductive devices [71]. Copyright 2023, *Nanoscale*. (**b**) The structure of the photoconductors. (**c**) Responsivity and internal quantum efficiency as a function of wavelength at 295 K with the bias = 60 V, The triangle represents internal quantum efficiency and the square represents responsivity [94]. Copyright 2014, *Advanced Functional Materials*. (**d**) Schematics illustrating the spray-coating setup and the device structures of phototransistors based on aqueous HgTe quantum dots (QDs) with varying QD film thicknesses achieved through multiple spray passes. The corresponding optical microscopy and SEM images of the QDs films are presented on the right [95]. Copyright 2017, *ACS Nano*. (**e**) Illustrations showing the device architecture of the P3HT:HgTe QD hybrid phototransistor. (**f**) Schematic diagram of the energy band alignment between P3HT and HgTe QDs. (**g**) Gate-voltage-dependent responses of a phototransistor based on HgTe CQDs and a phototransistor based on a P3HT:HgTe CQD hybrid. The responsivity at VGS = 0 V is used for comparison. The illumination level is 1550 nm, 22 mW cm^−2^. All data were acquired at room temperature [96]. Copyright 2020, *Adv Sci (Weinh)*.

Photoconductive devices are fabricated by directly depositing CQD films onto a substrate or dielectric layer with interdigitated electrodes. The typical device structure is shown in Figure 3a [71].

Keuleyan et al. prepared the first HgTe CQD photoconductor in 2011, which exhibited a photoresponse extending beyond 5 µm at room temperature. A detectivity of 2 × 10^9^ Jones at 130 K for a sample with a 5 μm cut-off was reported, which generated substantial research interest in HgTe CQDs as an affordable material system for IR photodetection [83]. In 2014, Chen et al. demonstrated a single-layer photoconductor structure based on aqueous HgTe CQDs with an ultrafast time response of up to 2 μs at 1600 nm [94] (Figure 3b,c). The device was fabricated using a simple spray-coating process and showed excellent stability in ambient conditions. The results exhibited that the gain and temporal response of aqueous HgTe-CQD-based photoconductors could be modulated by controlling the size and surface chemical properties of the CQDs. This provided a feasible method for optimizing photodetectors with optional sensitivity and operation bandwidth.

Using methods such as ligand exchange can enhance the material’s intrinsic properties, leading to an improvement in the detector’s performance. In 2019, Chen et al. introduced the novel approach of mixed ligand exchange. Compared with the previous “solid-state ligand exchange” CQDs using ethylene glycol, the new process increased the electron and hole mobility by a factor of 100, the response rate by a factor of 100, and the detectivity by a factor of 10. At 80 K, the detectivity reached 4.5 × 10^10^ Jones with a cut-off wavelength of 5 µm [89]. In a comparison of mid-infrared photodetectors, those fabricated using HgTe CQDs with novel hybrid mercaptoethanol-HgCl_2_ (ME-HgCl_2_) ligands were contrasted with those employing the conventional 1,2-ethanedithiol (EDT) surface treatment, and the authors found that the responsivity of the ME-HgCl_2_ ligand device increased by a factor of 50, reaching 0.23 A/W at 80 K [90]. After Liu et al. successfully synthesized I^−^ in situ passivated HgTe CQDs in 2022, they prepared a photoconductive device. By reducing the temperature, the device demonstrated exceptional detection capabilities under 2000 nm light; the noise current density of the photoconductive device was notably minimized to about 10^−11^ A/Hz^1/2^ at 130 K with the frequency of 1 Hz [92].

Phototransistors are another commonly used photodetector structure. Fabrication of typical phototransistors involves applying a coating of CQDs onto a silicon wafer that is affixed with a layer of silicon dioxide and patterned metal electrodes.

In 2013, Lhuillier et al. used the inorganic ligand As_2_S_3_ to passivate the HgTe CQDs via solid-film ligand exchange. They achieved a responsivity greater than 100 mA/W and a detectivity of 3.5 × 10^10^ Jones in transistor samples with a 3.5 μm cut-off at 230 K [86].

In order to improve the detectivity of the HgTe CQD photodetectors towards longer wavelengths, in 2017, to overcome this performance bottleneck, Chen et al. employed a synergistic approach combining synthetic chemistry and device engineering [95] (Figure 3d). Initially, they devised a fully automated synthesis method using an aprotic solvent and gas injection. This allowed for scalable fabrication of large-sized HgTe CQDs with high quality, showcasing a record-high photoluminescence quantum yield of 17% at 2080 nm. Subsequently, they achieved a HgTe-CQD-based phototransistor with a high specific detectivity of up to 2 × 10^10^ Jones in the 2000 nm wavelength range at room temperature utilizing a layer-by-layer spray-coating technique for the CQD layer. The achieved performance level was on par with that of commercially available room-temperature-operated, epitaxially grown photodetectors. In 2020, Dong et al. demonstrated a P3HT:HgTe QD hybrid phototransistor [96] (Figure 3e–g). By meticulously controlling the co-blend stirring and ligand exchange processes, the nanoscale morphology and charge transport of the hybrid layer were fine-tuned, leading to an optimized uniform phase distribution. Consequently, the device attained a specific detectivity exceeding 1 × 10^11^ Jones and a response time of less than 1.5 µs under room-temperature operation at 2400 nm.

In addition, photovoltaic devices feature a vertical multilayer geometric structure. Typically, CQD films serve as the photoactive layer sandwiched between the electron transport layer and the hole transport layer.

In 2015, Sionnest et al. pioneered the development of the initial HgTe CQD photovoltaic devices functioning in the mid-infrared, which achieved a specific detectivity of 4.2 × 10^10^ Jones and microsecond response times with a cut-off wavelength of 5.25 µm at 90 K [97]. This work has greatly stimulated the development of CQDs with fast, low-cost, and sensitive thermal infrared detection.

**Figure 4:**
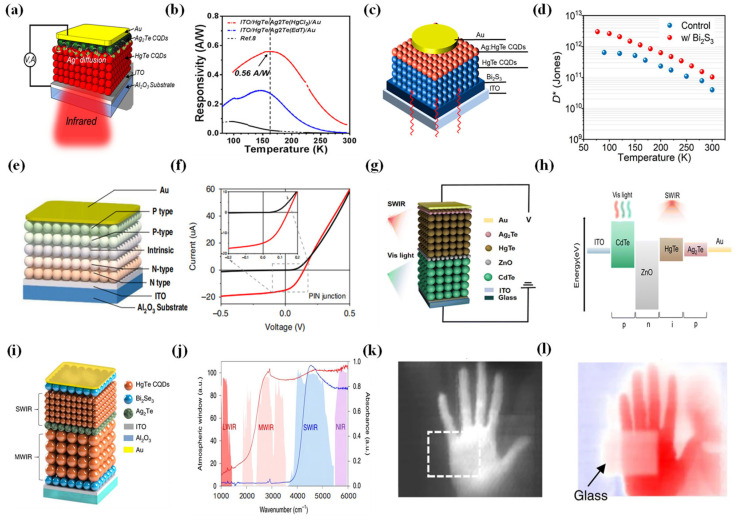
Photovoltaic devices using HgTe CQDs. (**a**) The common structures of photovoltaic infrared detectors. (**b**) The red circle represents the responsivity (T) of the HgTe CQDs MWIR detectors treated with HgCl_2_, whereas the black arrow represents the responsivity (T) of the HgTe PV MWIR detectors [100]. Copyright 2018, *ACS Nano*. (**c**) The device structure of HgTe CQD photodetectors with an added electron transport layer (ETL). (**d**) The blue line represents the relationship between the temperature and detectivity performances for the device without ETL, whereas the red line represents the relationship between the temperature and detectivity performances for the device based on Bi_2_S_3_ [101]. Copyright 2023, *ACS Photonics*. (**e**) The diagram illustrates the structure of a homojunction photodiode with high-mobility CQDs. (**f**) The I-V curve depicts a high-mobility PIN gradient homojunction device, with the inset focusing on the near-zero bias region at 80 K [93]. Copyright 2023, *Light Sci Appl*. (**g**) Structure diagram of the dual-band infrared detector. (**h**) Energy band structure diagram of the dual-band infrared detector [103]. Copyright 2023, *Journal of Materials Chemistry C.* (**i**) Depiction of a dual-band CQD imaging device structure with a bias voltage applied between the indium tin oxide (ITO) and the grounded Au contact. (**j**) The left axis represents the atmospheric transmission window, and the right axis indicates the optical absorption of SWIR and MWIR HgTe CQDs utilized in the fabrication of the dual-band device. The red line indicates HgTe CQDs with absorption characteristics in the MWIR region. The blue line indicates HgTe CQDs with absorption characteristics in the SWIR region (**k**) Using the detector to obtain SWIR images of a hand placed behind glass. The dotted line indicates the position of the glass. (**l**) Using the detector to obtain MWIR images of a hand placed behind glass [102]. Copyright 2019, *Nature Photonics*.

To realize high-performance optoelectronic devices, in 2018 Ackerman et al. demonstrated improvements in the sensitivity and efficiency of photovoltaic MWIR detectors by developing a p–n junction with enhanced light collection [98] (Figure 4a,b). They introduced a method of solid-state cation exchange, whereas the interface potential was chemically modified, leading to the improvement of the external quantum efficiency at room temperature by an order of magnitude. A sensitivity of 1 × 10^9^ Jones was achieved with a cut-off wavelength between 4 and 5 µm at 230 K. The responsivity was improved to 1.3 A/W at 85 K and the detectivity was improved to 3.3 × 10^11^ Jones with a cut-off wavelength of 5 µm. In 2022, Yang et al. developed a new p–i–n photodiode from the traditional p–i device structure [99] (Figure 4c,d). Bismuth sulfide (Bi_2_S_3_) films were adopted as the electron transport layer, which were beneficial to absorber deposition, superior charge extraction, and suppressed interfacial loss. The Bi_2_S_3_-based photodiodes showed a specific detectivity up to 1 × 10^11^ Jones at room temperature. Additionally, the Bi_2_S_3_-based photodiodes achieved a dark current density as low as 1.6 × 10^−5^ A/cm^2^ at −400 mV at room temperature.

The use of metal halide ligands can improve the photovoltaic performance [100] and enhance the photoluminescence of CQDs [101]. In 2020, Ackerman et al. introduced HgCl_2_ treatment to each layer of HgTe CQDs prior to the crosslinking with EDT/HCl [102]. The treatment with HgCl_2_ improves the R_0_A (the shunt resistance area product) by an order of magnitude and also slightly improves the responsiveness at room temperature. HgTe CQD photodiodes could achieve external quantum efficiencies of more than 50% at room temperature, detectivities of up to 1 × 10^11^ Jones at 2.2 μm, and response times at the microsecond level.

Achieving superior light detection often requires cooling down CQD infrared detectors due to the thermal carriers generated by a narrow mid-infrared energy gap. As a result, increasing the operating temperature of the detectors becomes crucial. In 2023, Xue et al. demonstrated a high-operating-temperature mid-infrared photodetector with a HgTe CQD gradient homojunction [91] (Figure 4e,f). The detector attained background-limited performance, exhibiting a specific detectivity of up to 2.7 × 10^11^ Jones at 80 K on 4.2 μm, exceeding 10^11^ Jones up to 200 K, surpassing 10^10^ Jones up to 280 K, and reaching 7.6 × 10^9^ Jones at 300 K for a wavelength of 3.5 μm. The external quantum efficiency also exceeded 77%, with a responsivity of 2.7 A/W at zero bias. Compared with single-spectrum detection, multispectral detection offers improved target recognition and enables the precise determination of the target’s infrared characteristics. Tang et al. used two different-sized HgTe CQD photodiodes stacked together to form a photodetector with dual-band detection in 2019 [103] (Figure 4g,h). By establishing stable spatial doping, a vertical stack of two rectifying junctions in a back-to-back diode configuration was created. The new device architecture allowed a bias-switchable spectral response between the short-wave infrared (SWIR) and the MWIR. This covered two critical atmospheric windows for infrared imaging. By manipulating the bias polarity and magnitude, the detector demonstrated rapid switching between short-wave infrared and mid-wave infrared at low temperatures, achieving modulation frequencies of up to 100 kHz and specific detectivities greater than 1 × 10^10^ Jones. Subsequently, Zhao et al. introduced an innovative dual-band detector utilizing CdTe and HgTe CQDs [104] (Figure 4i,j). This dual-band device, designed with precision, offered the capability to switch between the visible and the SWIR modes through adjustments in bias polarity and magnitude. The response peaks of the device were at 700 and 2100 nm in the visible and SWIR modes, respectively. Remarkably, this dual-band device exhibited exceptional performance metrics and featured a remarkably low noise current of approximately 10^−13^ A/Hz^1/2^ coupled with impressively high responsivity values of 0.5 A/W (VIS) and 1.1 A/W (SWIR). Moreover, it achieved a remarkable detectivity exceeding 10^11^ Jones in both the visible and SWIR modes, even when operating at room temperature.

To further enhance the performance of devices based on HgTe CQDs, the design of the device structure plays a pivotal role. The incorporation of structures such as plasmonic arrays and resonant cavities can effectively serve the purpose of improving detector performance.

**Figure 5:**
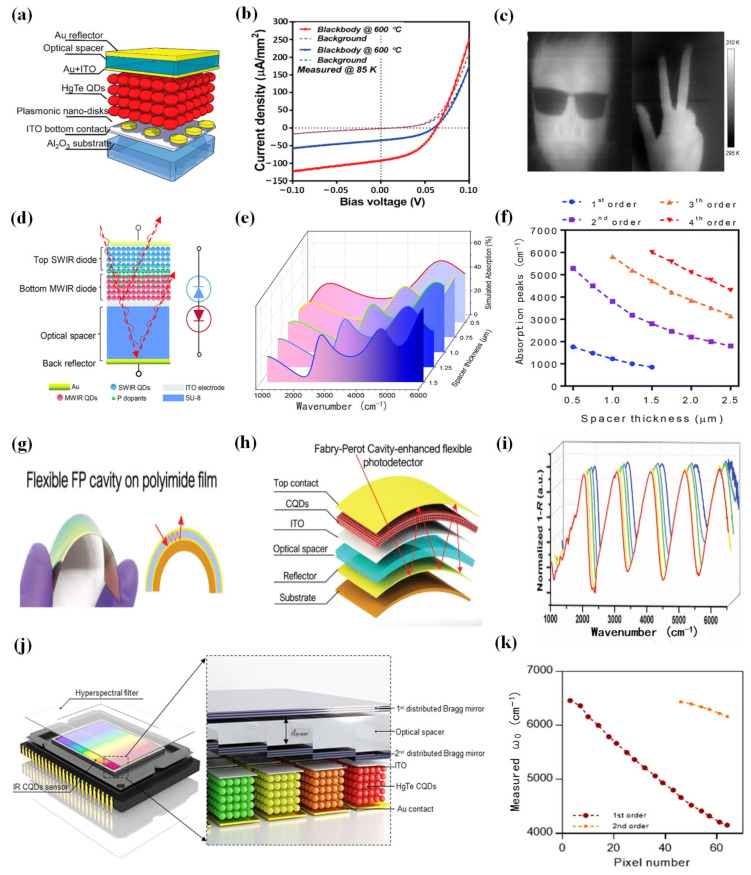
Microstructure HgTe CQD photodetection devices. (**a**) An illustration of the detector with an interference structure and plasmonic disk array based on HgTe CQDs. (**b**) The blue line represents the IV curve for the reference detector, whereas the red line depicts the IV curve for the detector based on 350 nm plasmonic disks. (**c**) When the MWIR HgTe-CQD-based detectors operated at 90 K, thermal images were captured [105]. Copyright 2018, *ACS Nano*. (**d**) The figure depicts the structure of a dual-band infrared detector based on CQDs. The equivalent circuit of the dual-band infrared photodiode is presented on the right. (**e**,**f**) Simulating the absorption for different thicknesses of the HgTe layer [106]. Copyright 2022, *Journal of Materials Chemistry C*. (**g**) The figure illustrates a flexible polyimide substrate Fabry–Perot cavity. (**h**) Schematic diagram of a HgTe CQD detector enhanced by a Fabry–Perot cavity. (**i**) The figure demonstrates measurements of “1—reflectance R” of the flexible Fabry–Perot cavity under different bending radii [107]. Copyright 2019, *Small*. (**j**) This image displays the internal structure of a CQD hyperspectral sensor. The sensor comprises a CQD sensor and hyperspectral filter. The optical spacers are positioned between two distributed Bragg reflectors and the CQD sensor array can be directly produced on the filter array. (**k**) The curve in the figure illustrates the function of peak responsivity (ω_0_) with respect to the pixel index [108]. Copyright 2019, *Laser & Photonics Reviews*.

In 2018, Tang et al. introduced a HgTe CQD photovoltaic detector that integrated HgTe CQDs and plasmonic structures and was aimed at enhancing the light absorption of the thin HgTe CQD layer [105] (Figure 5a–c). This integration resulted in a significant 2- to 3-fold improvement in responsivity at 5 μm, reaching an impressive 1.62 A/W. Moreover, this enhanced performance spanned a wide range of operating temperatures, from 295 to 85 K, achieving a detectivity of 4 × 10^11^ Jones at cryogenic temperatures. At an acquisition rate of 1 kHz for a 200 μm pixel, the noise equivalent temperature difference was measured at just 14 mK. Integrating a photodetector with a resonant cavity can enhance the light collection efficiency and spectral selectivity, thereby improving the overall performance of the detector. Luo et al. demonstrated the integration of a HgTe CQD dual-band infrared photodetector with a Fabry–Perot resonant cavity [106] (Figure 5d–f). This integrated photodetector exhibited an impressive responsivity of 1.1 A/W in the SWIR and 1.6 A/W in the MWIR. Notably, the detectivity was significantly enhanced, increasing by a factor of 2 and reaching up to 2 × 10^11^ Jones.

Flexible detectors and hyperspectral sensors have been demonstrated in a series of innovative applications. In 2019, Tang et al. successfully fabricated flexible HgTe CQD photovoltaic detectors and proposed a method to further enhance the light absorption in detectors by integrating a Fabry–Perot resonator cavity [107] (Figure 5g–i). Using this method, they prepared flexible infrared detectors with mechanical flexibility and high detectivity. Integrated short-wave IR detectors on flexible substrates had a peak detectivity of 7.5 × 10^10^ Jones at 2.2 µm at room temperature. In the same year, Tang et al. proposed the design of a CQD hyperspectral sensor by integrating a HgTe CQD photovoltaic sensor with a distributed Bragg mirror filter array [108] (Figure 5g,k). By directly integrating the CQD sensors with a distributed Bragg mirror filter array, 64 narrowband channels with full width at half maxima down to ≈30 cm^−1^ could be realized. The experiment effectively demonstrated high-resolution spectral measurements, achieving a resolving power of up to 180. Furthermore, it successfully acquired a hyperspectral image cube within the short-wave infrared range and benefitted from a swift response time of approximately ≈120 ns and an impressive sensitivity surpassing >10^10^ Jones that was exhibited by the CQD sensors.

FPA detectors can offer high spatial resolution. CQDs, in contrast to traditional bulk semiconductor materials in the infrared range, have significant advantages in FPA processing. They eliminate the need for complex molecular beam epitaxy processes and flip-chip bonding techniques, substantially reducing the manufacturing cost of infrared FPA detectors.

In 2016, Sionnest et al. presented the inaugural CQD FPA infrared detector designed for mid-infrared imaging. This was achieved by integrating HgTe CQDs with a silicon readout integrated circuit (ROIC) [109]. They showcased a straightforward fabrication approach for an economical HgTe CQD FPA thermal camera, achieving a noise equivalent differential temperature of 1.02 mK at 5 µm and capturing images at a rate of 120 frames per second.

**Figure 6:**
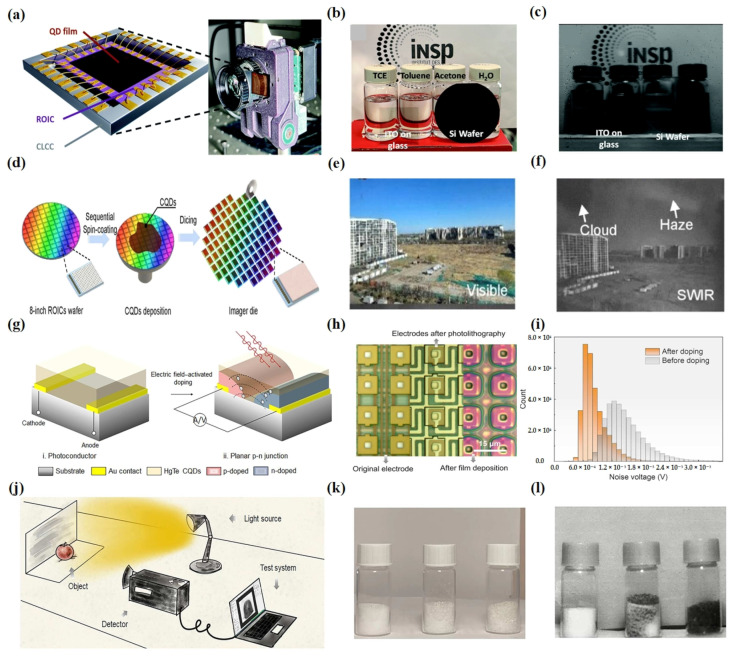
HgTe CQD focal plane arrays. (**a**) The figure shows the HgTe-CQD-based SWIR camera and the camera captures pictures with an M1614-SW objective. (**b**) A visible image captured using a smartphone camera depicting a scene with four vials arranged from left to right: tetrachloroethylene, toluene, acetone, and water. In front of the vials there is an ITO-covered glass slide and a two-inch silicon wafer. (**c**) Using an FPA based on HgTe CQDs to image the same thing as in (**b**) to obtain an SWIR imager [110]. Copyright 2022, *Nanoscale*. (**d**) The image provides a simple demonstration of CMOS-compatible manufacturing of CQDs with an ROIC. The wafer has an 8-inch diameter. Initially, the ROIC wafer undergoes a cleaning process, followed by spin-coating the CQD solution onto the wafer. Once the CQD thickness reaches the desired value, the 8-inch wafer is then cut into individual imaging sensor chips. (**e**) A visible image captured using a smartphone camera depicting a scene with sky and buildings. (**f**) Using an imaging sensor chip to image the same scene as in (**e**) to obtain an SWIR imager [112]. Copyright 2023, *ACS Photonics*. (**g**) Through in situ electric field activation doping, researchers successfully transitioned the working mode of the FPA detector from a photoducting mode to a planar pn photovoltaic mode, as schematically illustrated in the figure. (**h**) This image contains optical microscopic imaging of the substrate in three states. The leftmost depicts the original electrode, the middle part shows the electrode after photolithography, and the rightmost displays the electrode after deposition of quantum dots on the photolithographed electrode. (**i**) A comparison of the noise in the FPA imager before and after doping is presented in histogram form. (**j**) Illustration depicting the imaging device. (**k**) A visible image captured using a smartphone camera depicting a scene with three vials containing from left to right: salt, a mixture of salt and sugar, and sugar. (**l**) Using the FPA imager to image the same scene as in (**k**) to obtain an SWIR imager [69]. Copyright 2023, *Science Advances*.

Currently, most efforts on FPA research have focused on the device structure, which requires a multilayer deposition. The optimization of the design appears particularly crucial. Gréboval et al. achieved a photoconductive FPA based on HgTe CQDs [110] (Figure 6a–c). They demonstrated an FPA with a 15 µm pixel pitch presenting an external quantum efficiency of 4–5% (15% internal quantum efficiency) for a cut-off around 1.8 µm with Peltier cooling alone. HgTe-CQD-based FPAs operating in the photoconductive mode are prone to exhibiting larger dark currents. Subsequently, Alchaar et al. designed an FPA based on HgTe CQDs with photovoltaic operation [111]. They designed a diode stack compatible with a readout integrated circuit whose back-end processing was optimized to ensure compatibility with a complete diode stack deposition. The diode design was also optimized to generate a Fabry–Pérot cavity in which 50% of the light was effectively absorbed at the band edge. High-resolution images were achieved through the utilization of the optimized structure.

In 2023, Zhang et al. achieved a complementary metal oxide semiconductor (CMOS) compatible infrared device with trapping-mode photodetectors, in which the minority of carriers were trapped by a vertical built-in potential, contributing to both decreased dark current density and improved quantum efficiency [112] (Figure 6d–f). These trapping-mode CMOS imagers exhibited excellent photoresponse non-uniformity down to 4%, a dead pixel rate down to 0%, external quantum efficiency up to 175%, and detectivity up to 2 × 10^11^ Jones for SWIR (cut-off wavelength = 2.5 μm) at 300 K and 8 × 10^10^ Jones for MWIR (cut-off wavelength = 5.5 μm) at 80 K.

Subsequently, Qin et al. discovered through their research that uneven and uncontrollable doping methods, along with complex device configurations, limited the performance of FPA imagers in the photovoltaic mode [69] (Figure 6g–l). Consequently, they proposed a controllable in situ electric field activation doping method to construct lateral p-n junctions for simple planar-structured photodetectors based on SWIR HgTe CQDs. A flat p-n junction FPA imager with 640 × 512 pixels was successfully manufactured.

We summarize the performance of detectors based on HgTe CQDs in Table 1.

## 3. Research on HgSe CQDs

### 3.1. Synthesis of HgSe CQDs

HgSe CQDs are naturally n-doped in ambient conditions. They typically present several carriers per nanocrystal and consequently exhibit interband and intraband absorption in the mid-infrared range [113,114,115,116,117].

**Figure 7:**
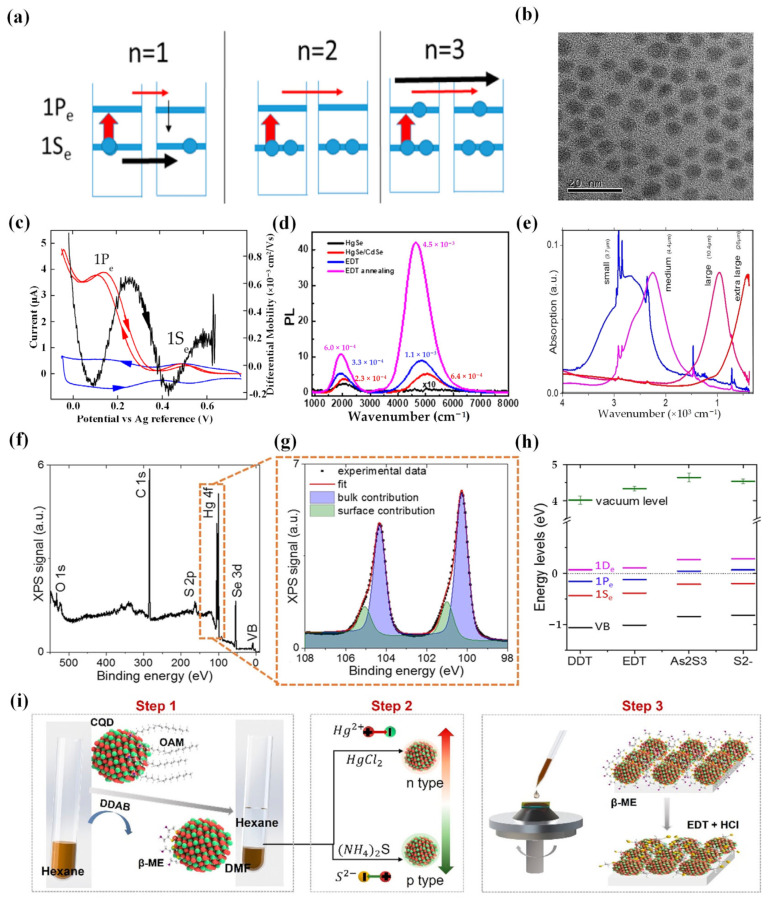
HgSe CQD characterization. (**a**) Band/intraband doping states of QDs. The red horizontal arrows represent photocurrent, and the black arrows represent dark current. The dark current is minimized when *n* = 2. (**b**) TEM image displaying HgSe CQDs. The particles exhibit an average diameter of 6.2 nm, and the standard deviation is 0.76 nm. (**c**) The blue line represents the Faradaic current, the red line represents the conduction current at a bias of 10 mV, and the black line represents the differential mobility of the HgSe CQD film on a platinum interdigitated electrode. The scan rate is 80 mV/s, and the electrolyte is tetrabutylammonium hexafluorophosphate in acetonitrile. The peaks at 0.5 V and 0.15 V in the reversible reduction/oxidation correspond to electron injection into the 1Se and 1Pe states, respectively. The conduction minimum at approximately 0.35 V signifies the filling of the 1Se state. The second minimum near 0 V indicates a conductivity gap between the 1Pe state and higher states. The arrows denote the scan direction [118]. Copyright 2014, *ACS Nano*. (**d**) PL spectra measured in air for HgSe/CdSe films subjected to different treatments [124]. Copyright 2017, *Chemistry of Materials*. (**e**) Absorption spectra of HgSe CQDs with different sizes. (**f**) Measurement of XPS signals for HgSe CQD film with an incident photon energy of 600 eV. (**g**) Photoemission spectrum of Hg 4f core-level emission in medium HgSe CQDs capped with As_2_S_3_. (**h**) Electronic spectrum representation (valence band in black, 1S_e_ state in red, 1P_e_ state in blue, 1D_e_ state in pink, and vacuum level in green) for medium-sized HgSe CQDs capped with four different ligands. The energy scale is referenced to the Fermi level and error bars indicate variations in the work function determined through different methods [127]. Copyright 2017, *ACS Appl Mater Interfaces*. (**i**) Illustration of the mixed-phase ligand exchange process. Step 1 involves liquid-phase ligand exchange. Step 2 includes doping modification using additional salts. Step 3 consists of solid-phase ligand exchange [130]. Copyright 2022, *ACS Nano*.

In 2014, Deng et al. conducted a study on the photoconductivity of doped HgSe CQDs with intraband transitions [118] (Figure 7a–c). HgSe CQDs also exhibited intraband photoluminescence. Compared with the interband transitions of CQDs, intraband transitions provided a selective spectral detection, which opens up the possibility for more materials to be applied to infrared applications.

The intraband carrier lifetime is vital in device applications, which are mainly limited by nonradiative processes and a low photoluminescence quantum yield. A core/shell nanocrystal structure can significantly enhance photoluminescence [119,120,121,122] and improve the chemical and thermal stability of materials. The shell can passivate surface states of the core, reducing non-radiative recombination pathways. Based on this, in 2015, Deng et al. synthesized HgSe/CdS core/shell CQDs and investigated the changes in their optical absorption and emission [123]. The intraband photoluminescence of HgSe/CdS films was higher than for HgSe films. However, the shell greatly improved the film’s resistance. After annealing at 200 °C, HgSe/CdS films maintained narrow intraband emission and higher laser power at a wavelength of 5 μm. In 2018, Shen et al. synthesized HgSe/CdSe core/shell CQDs, achieving the brightest emission at 5 μm, with a quantum yield of approximately 10^−3^ [124] (Figure 7d).

In 2021, Kamath et al. conducted an in-depth study of the core–shell structure [125]. They reported the synthesis and spectral analysis of thick-shell n-type HgSe/CdS core/shell CQDs. Under the milder conditions, HgSe/CdS CQDs with thick shells were synthesized via a two-step growth process utilizing highly reactive single-source precursors. It was found that the intraband lifetime and photoluminescence quantum yield increased with an increase in shell thickness. The maximum photoluminescence efficiency and longest intraband lifetime were achieved at 2000 cm^−1^. This is the brightest solution phase mid-infrared chromophore reported to date, achieving an internal photoluminescence quantum yield of 2%. Additionally, this photoluminescence had an intraband lifetime of over 10 ns. Later in 2023, Shen et al. presented electroluminescence at 5 µm by utilizing the intraband transition between the 1S_e_ and 1P_e_ states located within the conduction band of core–shell HgSe–CdSe CQDs [126].

To boost the performances, i.e., reducing dark current and improving the time response of the HgSe CQDs, Martinez et al. determined the electronic spectrum on an absolute energy scale for HgSe CQDs with different sizes and different ligands in 2017 [127] (Figure 7e–h). Subsequently, they introduced a technique involving the grafting of functionalized polyoxometalates (POMs) onto the surface of HgSe CQDs. This approach resulted in a substantial tuning of the carrier density (approximately five electrons per nanoparticle) and conduction properties simultaneously [128]. This method showed great promise for achieving significant tuning of the carrier density in degenerately doped semiconductor nanoparticles.

In order to further improve the mobility of CQDs, Chen et al. proposed a method of hybrid ligand exchange (HgSe/hybrid) to prepare the HgSe CQD film and then compared it with the solid-state ligand exchange of ethanedithiol (HgSe/EDT) [129]. In contrast, the mobility of HgSe/hybrid films showed a 100-fold increase in mobility, reaching 1 cm^2^/Vs for particles with a diameter of 7.5 nm. Subsequently, they introduced a room-temperature mixed-phase ligand exchange method that allowed for air-stable doping between the 1S_e_ and 1P_e_ states of high-mobility HgSe CQDs that could be adjusted [130] (Figure 7i). Moreover, Chen et al. explored how the size distribution of intraband HgSe CQD films impacts transport and photodetection [131]. The results indicated that enhancing the uniformity of HgSe CQD sizes leads to a significant increase in mobility that correlates with the energy distribution resulting from this size consistency.

### 3.2. HgSe CQD Photodetectors

**Figure 8:**
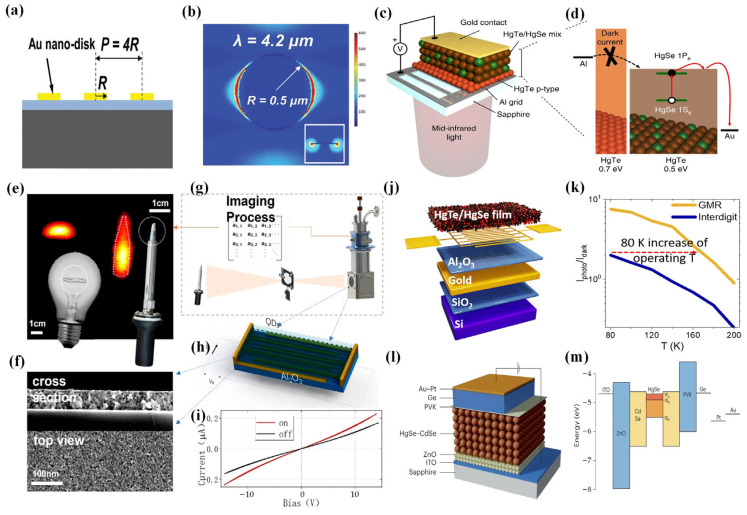
HgSe CQD photodetector. (**a**) The illustration shows a visual depiction of plasmonic disk arrays situated on a SiO_2_/Si substrate. (**b**) The graphic illustrates the simulated enhancement ratio corresponding to the resonance wavelength. The inset provides a detailed cross-sectional view for a more comprehensive understanding [132]. Copyright 2017, *Journal of Materials Chemistry C*. (**c**) The device structure. Lighting is supplied from the device’s rear, with the sapphire substrate enabling a 70% transmission of mid-infrared light. (**d**) The band diagram illustrates the alignment for the diode structure. HgTe 6k functions as a unipolar barrier, effectively filtering the injection of dark current into the active HgSe/HgTe 4k layer [135]. Copyright 2019, *Nat Commun*. (**e**) Infrared thermal image obtained with a HgSe intraband CQD photodetector. (**f**) SEM of top view and cross section of a photoconductor based on HgSe CQDs. (**g**) Diagram of the imaging scanning device. (**h**) Schematic diagram of a photoconductor based on HgSe CQDs. (**i**) The current of the HgSe-CQD-based photoconductor is depicted by the black line in the absence of blackbody radiation, whereas the red line represents the current in the presence of blackbody radiation. The effective surface area is approximately 0.3 mm × 0.16 mm [130]. Copyright 2022, *ACS Nano*. (**j**) The diagram illustrates the GMR (giant magnetoresistance) device integrated with an intraband absorbing film composed of the HgSe/HgTe nanocrystal mixture. (**k**) The variation in the intraband photocurrent (induced by QCL illumination at 4.4 μm) to dark current ratio is depicted as a function of temperature. This comparison is made between reference interdigitated electrodes (without resonance) and GMR electrodes [136]. Copyright 2022, *ACS Photonics*. (**l**) Structure of the device. (**m**) Energy band diagram of the device [126]. Copyright 2023, *Nature Photonics*.

As HgSe CQD synthesis technology has gradually matured, researchers have delved into the study of photodetectors based on HgSe CQDs. In 2017, Tang et al. prepared a kind of plasmonic nanodisk array narrowband photodetector by using HgSe CQD films as narrowband light absorption sensing materials [132] (Figure 8a,b). The responsivity at the center wavelength of the narrowband detector was improved by integration with the plasmonic nanodisk array. This was the first time that plasmonic nanodisk arrays had been integrated into MIR detectors based on HgSe CQDs. The results showed that the responsivity at the center wavelength increases significantly and that the full width at half maximum (FWHM) values of the photodetectors decreases. This work was of great significance for the development of filter-free narrowband infrared detectors and cameras operating at room temperature.

Modifying HgSe CQDs hinges significantly on the selection of appropriate ligands. Notably, hybrid ligand exchange has made substantial advancements in achieving tunable doping and high mobility in interband CQDs. However, research on intraband CQD detectors is still scarce. Therefore, Chen et al. developed a high-performance photodetector in 2022 [130] (Figure 8e–i). This photodetector could serve as an intraband infrared camera for thermal imaging, as well as a CO_2_ gas sensor with a range from 0.25 to 2000 ppm. A relatively high carrier mobility (1 cm^2^/Vs) in HgSe intraband CQD solids was obtained by utilizing a room-temperature mixed-phase ligand exchange method. Furthermore, the high mobility and controllable doping proved beneficial for a mid-infrared photodetector utilizing the 1S_e_ to 1P_e_ transition, with a 1000-fold improvement in response speed, which was several μs, a 55-fold increase in responsivity, which was 77 mA/W, and a 10-fold increase in detectivity, which was above 1.7 × 10^9^ Jones at 80 K. In 2022, Sokolova et al. compared the effects of four different types of ligands (EDT, BeSH, S^2−^_,_ and SCN) on the sensitivity of infrared photodetectors based on HgSe CQDs [133]. The maximum sensitivity of the photodetectors was obtained when SCN was used for the ligands. The obtained responsivity and detectivity of the photodetectors were 0.5 A/W and 3.1 × 10^7^ Jones, respectively.

By mixing with other materials, it is possible to overcome the inherent flaws of the material itself while preserving its original advantages, thus enhancing the performance of the detector [134]. In 2019, Livache et al. proposed a design for a CQD infrared photodetector metamaterial from a mixture of HgSe and HgTe CQDs [135] (Figure 8c,d). At the same time, they integrated this material into a photodiode and achieved a detectivity of 1.5 × 10^9^ Jones at 80 K, which was two folds higher than HgSe CQDs operating at the same temperature and wavelength. In 2022, Khalili et al. proposed a dye-sensitization method to overcome the limitations (high dark current, slow response, low activation energy) observed from internal materials. They used a mixture of HgSe and HgTe as a dye-sensitized infrared sensor [136] (Figure 8j,k). This hybrid material maintained the internal absorption of HgSe CQDs while reducing the dark current, increasing activation energy, and fixing time response. They also studied carrier dynamics in the material using infrared transient spectroscopy and measured the coupling between the two materials. On this basis, they proposed a strategy to improve the optical detection performance of hybrid materials by coupling internal absorption. The mixed material was coupled with a guided mode resonator. The detectivity reached 10^9^ Jones at 80 K and the responsivity reached 3 mA/W with a weak temperature dependence.

The design of a core–shell structure can enhance the performance of a photodetector by improving the intrinsic properties of the material itself. In 2023, Shen et al. designed a core–shell structure core–shell HgSe–CdSe CQDs [126] (Figure 8l,m). They applied this material in a photodiode; the device exhibited an external quantum efficiency (EQE) of 4.5% at 2 A/cm^2^, with a power efficiency of 0.05%. This 4.5% EQE was comparable with that of commercial epitaxial cascade quantum well light-emitting diodes.

Based on the above work, it can be seen that effective ligand exchange, mixing with other materials, and the design of the core/shell structure are all strategies for improving device performance.

We summarize the performance of detectors based on HgSe CQDs in Table 2.

## 4. HgS-CQD-Based Photodetectors

HgS CQDs are a kind of quantum dots with strong constraint; under ambient conditions, the carriers remain stable in the lowest conduction band state [137].

**Figure 9:**
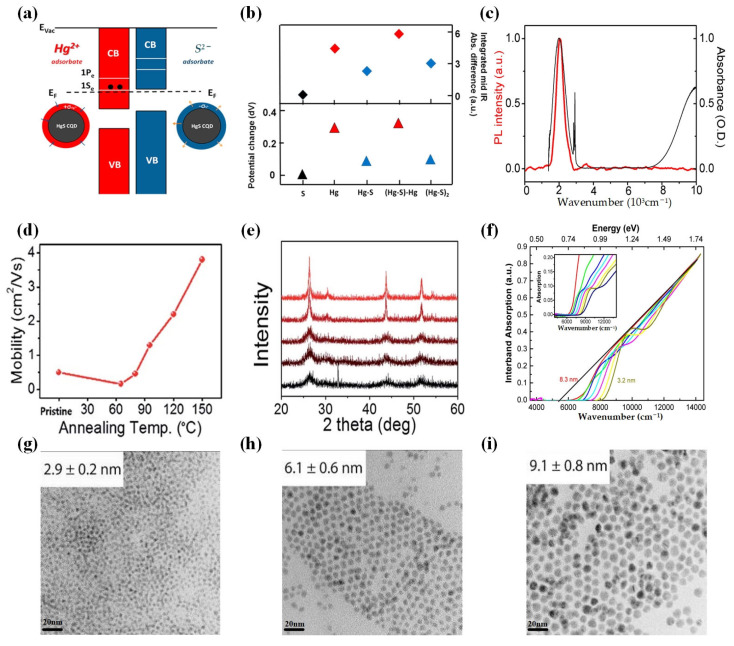
HgS CQD characterization. (**a**) Energy diagrams for Hg^2+^-doped and S^2−^-treated HgS CQDs are depicted schematically. (**b**) Relative to the initial measurement of the ethanedithiol cross-linked HgS CQD film, this presentation includes the resting potential difference and the integrated mid-infrared absorption of a HgS colloidal quantum dot (CQD) film. These measurements were taken after alternate exposure to Hg^2+^ and S^2−^ ions, with the resting potential being assessed 40 s after immersion in the electrolyte. (**c**) After photoexcitation at 808 nm, the intraband photoluminescence emission (depicted in red) arises from the 1S_e_–1P_e_ transition of ambient n-type HgS nanocrystals. The photoluminescence emission spectrum (in red) is overlaid with the absorption spectrum (in black) [138]. Copyright 2014, *J Phys Chem Lett*. (**d**) As a function of annealing temperatures, the linear mobility of HgS CQDs thin-film transistor devices is plotted. (**e**) The XRD spectra of HgS CQDs at the different temperatures are presented [139]. Copyright 2017, *RSC Advances*. (**f**) Interband absorption post-sulfide treatment of CQDs with different size, normalized at high energy, is presented, as measured in solution. The solid black line represents an extrapolation of the absorption at higher energies, with the x-intercept serving as an estimate for the bulk gap, measured at 0.67 eV. (**g**–**i**) TEM images of HgS CQDs with different sizes spanning from 2.9 to 9.1 nm [140]. Copyright 2016, *The Journal of Physical Chemistry C.*

In 2014, Jeong et al. conducted a comprehensive investigation on HgS CQDs [138]. They demonstrated, for the first time, CQDs with stable electron occupancy in the lowest quantum state under ambient conditions, as well as the emergence of intraband luminescence. Figure 9 characterizes the properties of HgS CQDs.

**Figure 10:**
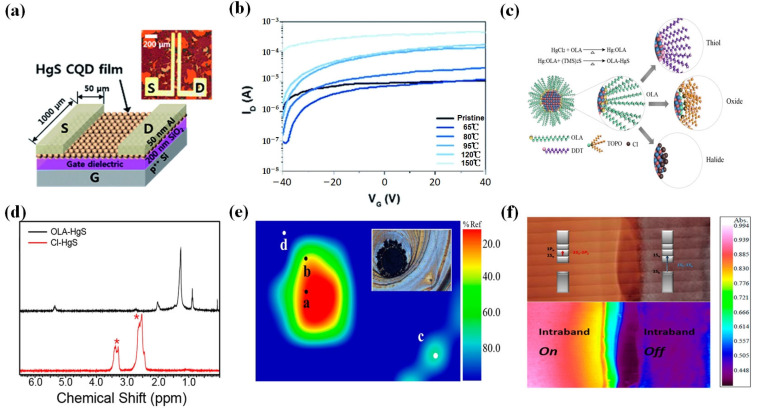
HgS CQD modifications. (**a**) The TFT device structure of HgS CQDs is schematically illustrated, along with an optical image. (**b**) Transfer characteristics of HgS CQDs TFTs, with annealing temperatures ranging from 65 to 150 °C, are displayed [139]. Copyright 2017, *RSC Advances*. (**c**) Versatility in ligands for oleylamine-passivated HgS CQDs is demonstrated, transitioning from amine to thiol, amine to oxide, and amine to halide atomic ligands. (**d**) The bottom spectrum (in red) represents the 1H NMR spectrum of ammonium-chloride-passivated HgS CQDs, indicating the absence of residual oleylamine ligands except for solvent peaks (marked with a star). For reference, the top spectrum corresponds to the NMR spectrum of oleylamine-passivated HgS CQDs. (**e**) The FTIR image of the identical solid HgS CQDs is presented, with an optical image provided in the inset. This figure is obtained by FTIR microscope. The point a and b are two different absorption peaks of the film. (**f**) The electron doping density of two distinct ligand-passivated HgS CQD solid films is monitored using an FT-IR microscope. The top part of the image displays the optical view, whereas the bottom part presents the FTIR image. In the bottom left-hand area (red–yellow), a heavily doped HgS CQD film exhibits mid-IR intraband absorption at 2275 cm^−1^. Conversely, the bottom right-hand area (blue–purple) represents a sulfide-treated HgS CQD film showing no mid-IR intraband absorption. The film geometry resembles a p-n junction. The image size is 8356 × 700 μm^2^ [141]. Copyright 2016, *Journal of Physical Chemistry C*.

Based on the examination of the electron occupancy of the semiconductor conduction band’s lowest electronic state (1S_e_), Kim et al. further investigated the electron mobility of HgS CQDs using field-effect transistors [139] (Figure 10a,b). The optimum carrier mobility was obtained by optimizing annealing temperatures and efficient ligand exchange. Finally, the measured mobility reached an impressive value of 1.29 cm^2^/Vs.

To further enhance the performance of HgS CQDs, researchers have proposed an innovative approach for the growth of CQDs. To address the issues of particle aggregation and poor shape control (size distribution) for HgS CQDs, in 2016, Shen et al. utilized a dual-phase method to synthesize HgS CQDs at room temperature, resulting in HgS CQDs with small size dispersion and well-defined optical features [140]. They also employed the same synthetic method to create HgS/CdS core/shell structures. The findings revealed that encapsulating HgS within a CdS shell effectively eliminated the natural n-doping of the HgS core, leading to interband photoluminescence at 1.5 μm with a quantum yield of approximately 5%. Additionally, the core/shell structure significantly enhanced the thermal stability of the HgS core.

The strong binding strength between thiol and HgS CQDs makes it difficult for HgS CQDs to exchange with other ligands species. To solve this problem, Yoon et al. employed the oleamine passivation method to synthesize HgS CQDs, offering a novel approach for producing thiol-free nanocrystals with electrons occupying the lowest quantum state of the conduction band [141] (Figure 10c–f). They achieved heavy doping of HgS CQDs without thiol ligands for the first time, effectively simplifying the process of ligand exchange. Non-thiol ligand-passivated HgS CQDs exhibit strong steady-state band-to-band transitions under ambient conditions. This work provided a direction for the erasable storage, infrared optoelectronics, and infrared free-space optical communication of CQDs.

At present, there is not much research on HgS CQD infrared detectors. The stability HgS CQDs needs to be further improved.

## 5. Conclusions

Mercury chalcogenide quantum dots, owing to their tunable bandgap, high absorption coefficients, and cost-effective solution processability, are considered promising materials for IR photodetectors. This article provides a comprehensive overview of the research progress in mercury sulfide quantum dot photodetectors, covering aspects such as quantum dot synthesis, material enhancements, device structures, and operating principles. Comprehensive analysis of the detector performance based on these materials reveals several key insights. Compared with other materials, detectors based on HgTe CQDs demonstrate a broader wavelength coverage and relatively superior performance. However, their stability is comparatively lower. HgSe CQDs can leverage intraband transitions for photoelectric detection. In contrast to the inter-band transition in HgTe CQDs, the intraband infrared luminescence attenuation exhibits little or greatly reduced Auger relaxation. This phenomenon arises from the sparse state density in the conduction band. Consequently, the utilization of intraband transitions proves advantageous for enhancing the quantum efficiency of infrared detectors. Nevertheless, achieving precise control over the size and electronic doping state of HgSe CQDs presents a significant challenge. Consequently, the yield of HgSe CQD detectors is not high and their performance lags behind that of HgTe CQD detectors. HgS CQDs are currently hampered by issues such as particle aggregation, limited shape control, and instability in preparation, resulting in a relatively sparse body of related studies. Nonetheless, the ongoing development of new technologies, including core–shell structures, holds the promise of addressing these challenges and enhancing the application potential of HgS CQDs in the field of detectors.

Over the past decade, significant breakthroughs have been achieved in the field of colloidal mercury sulfide quantum dots, ranging from single-point detectors to focal plane arrays. However, the colloidal mercury sulfide quantum dot system still faces several challenges:

1. Material Stability: Mercury sulfide colloidal quantum dots may undergo degradation or deterioration when exposed to prolonged use or high-temperature environments, limiting their widespread applications. Surface modification of quantum dots with organic or inorganic ligands is essential to enhance their stability and improve their dispersion in solutions. This is crucial for the preparation of long-term stable and reliable infrared detectors.
2. Limited Photodetection Range: Mercury sulfide quantum dots are primarily used in the near-infrared, short-wave infrared, and mid-infrared spectral range, with fewer applications in the long-wave infrared range. Expanding their photodetection range is necessary to achieve broader spectral detection capabilities.
3. Operating Temperature: Theoretically, the quantum-mechanical nature of CQDs implies higher operating temperatures for IR photodetectors, since thermal carrier generation would be significantly reduced compared with the quantum well due to the energy quantization in all three dimensions. Still, cooling is necessary in most CQD photodetectors. Further theoretical and experimental research is needed.

## 6. Outlook

Mercury chalcogenide CQDs for infrared detectors hold immense promise for future development. Firstly, the simplified fabrication method based on solution manufacturing significantly reduces production costs. Secondly, infrared detectors based on mercury chalcogenide CQDs, with their tunable bandgap and extensive absorption spectra, offer a broader spectral range, making them potentially applicable in various fields. Prospective applications may span biomedical imaging, communication technology, solar cells, and more. Additionally, the flexibility of mercury chalcogenide CQDs in terms of compatibility, allows for application on various substrates, particularly silicon and flexible substrates. Through optimizing synthesis methods, improving material stability and designing novel device structures, achieving higher EQE and faster response times is possible. In summary, mercury-chalcogenide-CQD-based infrared detectors, with their unique properties, demonstrate extensive prospects for applications such as infrared imaging technology, solar cells, biomedical imaging, gas sensing, and environmental monitoring.

## Figures and Tables

**Table 1:** The summary of photodetectors based on HgTe CQDs.

Year	Photoactive Material	Detection Range (nm)	Detectivity (Jones)	Responsivity (A/W)	Rise Decay Time	Ref.
Photodetectors based on HgTe CQDs						
2011	HgTe CQDs	5000	2 × 10^9^	--	--	[83]
2013	HgTe CQDs/As_2_S_3_	3500	3.5 × 10^10^	0.1	--	[86]
2014	HgTe CQDs	1600	--	--	2 μs	[94]
2015	HgTe CQDs	5250	4.2 × 10^10^	--	--	[97]
2016	HgTe CQDs	3600	2 × 10^10^	--	--	[109]
2017	HgTe CQDs	2000	2 × 10^10^	0.4	12.6 μs	[95]
2018	HgTe CQDs	4000/4500	1 × 10^10^ @4000 nm 4 × 10^10^ @4500 nm	1.62 @4500 nm	--	[105]
2018	HgTe CQDs/Ag_2_Te	5000	3.3 × 10^11^	1.3	--	[98]
2019	HgTe CQDs	5000	5.4 × 10^10^	--	--	[89]
2019	HgTe CQDs	2200	7.5 × 10^10^	--	--	[107]
2019	HgTe CQDs	2500/4500	1 × 10^10^	--	--	[103]
2020	HgTe CQDs	4000	5.4 × 10^10^	0.23	--	[90]
2020	HgTe CQDs/P3HT	2400	>1 × 10^11^	--	<1.5 μs	[96]
2022	HgTe CQDs	2350/4000	2 × 10^11^	1.1 @2350 nm 1.6 @4000 nm	--	[106]
2023	HgTe CQDs	3500/4200	7.6 × 10^9^ @3500 nm @300 K 2.7 × 10^11^ @4200 nm @80 K >1 × 10^11^ @4200 nm @200 K >1 × 10^10^ @4200 nm @280 K	2.7	--	[91]
2023	HgTe CQDs/Bi_2_S_3_	2200	1 × 10^11^	--	8 μs	[99]
2023	HgTe CQDs/CdTe CQDs	700/2100	1 × 10^11^ @700 nm 4.5 × 10^11^ @2100 nm	0.5 @700 nm 1.1 @2100 nm	--	[104]
2023	HgTe CQDs	2500/5500	2 × 10^11^ @250 nm 8 × 10^10^ @5500 nm	--	--	[112]

**Table 2:** The summary of photodetectors based on HgSe CQDs.

Year	Photoactive Material	Detection Range (nm)	Detectivity (Jones)	Responsivity (A/W)	Rise Decay Time	Ref.
Photodetectors based on HgSe CQDs						
2014	HgSe CQDs	5000	8.5 × 10^8^	5 × 10^−4^	--	[118]
2017	HgSe CQDs	4200/6400/ 7200/9000	--	0.145 @4200 nm 0.092 @6400 nm 0.088 @7200 nm 0.086 @9000 nm	--	[132]
2019	HgSe CQDs/HgTe CQDs	4400	1.5 × 10^9^	--	<500 ns	[125]
2022	HgSe CQDs	4000	1.7 × 10^9^	0.077	--	[131]
2022	HgSe CQDs	--	3.1 × 10^7^	0.5	--	[134]
2022	HgSe CQDs/HgTe CQDs	5000	1 × 10^9^	0.003	<200 ns	[136]

## Data Availability

Data are contained within the article.
